# Anti-racism Training Using the Biopsychosocial Model: Frederick Douglas' Earthquake, Whirlwind, Storm and Fire

**DOI:** 10.3389/fpsyt.2021.711966

**Published:** 2021-10-05

**Authors:** Mechelle Sanders, Kevin Fiscella

**Affiliations:** Department of Family Medicine, Highland Family Medicine Research, University of Rochester Medical Center, Rochester, NY, United States

**Keywords:** biopsychosocial approach, racism, medical education, communication, physician-patient relations, community advocacy

## Abstract

Rochester, New York is home to George Engel and the Biopsychosocial (BPS) model. Rochester was also home to Fredrick Douglas and a stop on the Underground Railroad. More recently, Rochester, New York is also where Daniel Prude died at the hands of the police. In this article, we discuss how our department of family medicine has incorporated race and racism into the BPS model and how we have used it to help primary care trainees become more effective in their work with Black Indigenous and people of color (BIPOC) patients.

## Introduction

The University of Rochester is across the street from the grave sites of two historical leaders for social justice in the United States: Frederick Douglass (1) and Susan B. Anthony (2, 3). Both were internationally renowned change agents for the disenfranchised. Douglass was an abolitionist and talented orator who not only spoke out against slavery, but also risked his life to lead Rochester's hub for the Underground Railroad that passed through his Rochester farmhouse. Susan B. Anthony was a leader in the women's suffrage movement, an anti-slavery activist and a lifelong friend of Douglas.

The University of Rochester, was once a professional home for George Engel and Harriet A. Washington. In 1977, Dr. Engel proposed the need for medicine to shift from an exclusive biomedical focus to a new model of care that integrates psychosocial elements of people's lives–A Biopsychosocial (BPS) model (4). The BPS model takes into account the patient, the social context in which they live and the clinician's role and the health care system in which they operate. Almost 30 years later, Washington won the National Book Critics Circle Award (5) for *Medical Apartheid: The Dark History of Medical Experimentation on Black Americans from Colonial Times to the Present*, a historical account of the racist travesties of medical experimentation on African Americans (6).

More recently, Rochester is known for the 2020 death of Daniel Prude (7). Mr. Prude suffered from mental illness. He was seen at the University of Rochester emergency department for self-injurious behavior and released. He died after being physically restrained by police officers, the cause of death was determined by the coroner to be homicide (8). In response to the events surrounding his death, medical students at the University of Rochester indicated that “Not only do our current models of healthcare leave gaping holes for individuals such as Daniel Prude to fall through, but they do so in manners which are fraught with racism (9)”.

The BPS model has transformed how medical educators teach trainees how to talk with patients (10, 11), but does not directly address the role of racism in the lives of Black, Indigenous and people of color (BIPOC) patients. Rather than asking a series of closed ended or narrow questions designed to quickly make a diagnosis, George Engel urged clinicians to listen while patients told their story. Engel insisted that more often than not, patients' uninterrupted history would provide key, psychosocial contextual factors that would enable the clinician to not only understand their patients' illness within context, but also provide guidance for more effectively treating the patient. Yet racism was not part of Engel's model.

To better understand the role of racism, we turn to the words of Fredrick Douglass. Using his words to build a framework for incorporating racism into the BPS model for teaching and praxis:

“*For it is not light that is needed, but fire; it is not the gentle shower, but thunder. We need the storm, the whirlwind, and the earthquake”*.

*- Frederick Douglass during a Fourth of July celebration in Rochester, NY in 1852* (12).

## Discussion

### The Thunder

Many White trainees have learned to be “color blind” and to treat people the same (13, 14). White culture following the Civil Rights legislation has tended to minimize the role of racism on the lives of BIPOC often resulting in White denial of everyday racism and much less one's own implicit racial biases or one's own privilege based on White skin color (15–18). The stream of viral videos documenting violence toward BIPOC has helped weaken the White taboo about discussing racism. It has created an opening for dialogue, if not the thunder and lightning bolt, regarding the profound impact it has on the lives of BIPOC and on the psyches of Whites (19).

White denial of racism can be addressed through activities such as implicit bias training, self-reflection and creating psychological safety in which trainees can share their own experiences with discrimination, stigmatization and marginalization (20). These discussions are not intended to equate experiences, but rather to sensitize trainees to their own affective experiences. Having skilled, racially diverse facilitators can increase psychological safety, particularly when facilitators can acknowledge and role model, sharing what they have learned about their own blind spots and implicit biases. This “gentle shower” prepares trainees for the thunder of BIPOC patient experiences. Recognition of racism and privilege can sensitize trainees to the role of the micro-dynamics of power within the patient-clinician relationship in addition to the macro dynamics of power and privilege in society including how it shapes politics, structural social disadvantage and constrained opportunities and privilege in education, law, employment and health care.

### The Storm

For trainees who have not talked about race or racism with patients, it may feel like entering a storm of emotions. Patients may recount trauma, anger, sadness and despair related to racism whether structural, interpersonal, or even internalized. Like storms, emotions can ebb and surge unexpectedly. Trainees may feel they are losing control of the visit. And trainees accustomed to intervening will struggle to listen and witness patients' experiences without attempting to rationalize them. Trainees often need reassurance that listening and witnessing is more powerful by itself. Engel taught his trainees that careful listening offers insights into the patient's biopsychosocial context. When a racism lens is added, trainees learn how race affects the life of the patient, while providing an informal lesson in racism based on mini-ethnography (21).

### The Whirlwind

Some trainees will struggle with the whirlwind of their own emotions. Some will need coaching in avoiding premature or false re-assurance. Others will need to be coached in channeling their own righteous anger. Others may need to seek out counseling for themselves when these experiences trigger their own past traumas and feelings of being marginalized or degraded. This whirlwind of emotions becomes an opportunity to teach trainees mindfulness related to the emotions expressed by patients, but most importantly acknowledging their own emotions and how to effectively manage them during patient visits (22).

### The Earthquake

Many trainees may experience an earthquake that shakes up their own assumptions about racism in the world, its impact on their own privilege and its pernicious impact on their own implicit attitudes. Skilled facilitation of diverse anti-racism groups in which members can be vulnerable provide space for members to reflect on racism and its impact on themselves and colleagues. Time devoted to sharing of experiences and reflections can create added safety.

### The Fire

Earthquakes and whirlwinds are often accompanied by fire. It was fire that Douglas was seeking to ignite among his White audiences, notably his “What to the Slave Is the Fourth of July” speech in Rochester in 1852 (23). Douglas hoped that this internal fire would inspire his audience to take up action against racism and its manifestation in chattel slavery. Similarly, many trainees will experience a fire to act. We have found this can be channeled through advocacy. Advocacy can be patient-based. This can mean connecting patients with resources to address social determinants of health (24). It can also mean advocating for patients within the health care system while supporting the patients' voice (25). It also means partnering with organizations to address structural racism (26). Our department sponsors a monthly seminar that includes documentaries, e.g., the Rochester race riot and speakers who address ways that trainees can take action. These sessions can help trainees find opportunities for advocacy, whether serving on antiracism departmental or medical school committees, supporting and empowering minoritized group organizations, or working on health equity, quality improvement initiatives.

The tragic death of Daniel Prude offers a powerful and heartrending story for discussion of how structural, interpersonal and internalized racism affects patients. According to press accounts (27), Mr. Prude's life was all too familiar. He was a father of five children who lived in Chicago where he grew up in a public housing complex. Two of his siblings died in tragic incidents that traumatized him. As an adult, Mr. Prude worked in warehouses and factories on the Southwest side of Chicago, while helping other people in his neighborhood get jobs. In 2018, a nephew of Mr. Prude committed suicide by gunshot in the home they shared. After this trauma, Mr. Prude reportedly, increasingly used phencyclidine (PCP) with resulting erratic behavior. He took his final train trip to Rochester, NY after his sister told him to leave her home due to his growing paranoia. Once in Rochester, his brother took him to the hospital for erratic behavior where he calmed down. He was subsequently released, only to run away from his brother's home before dying, hooded, naked, and restrained by police, who reportedly applied pressure under his jaw to a nerve, pinning him to the street. The narrative and 11-min police video painfully underscore how structural, interpersonal, and internalized racism interacted with the psychosocial context of Daniel Prude's life–ultimately ending it with a half dozen police in witness.

Medical trainees should be encouraged to “light fires” and engage in civic action in order to prevent and dismantle racist policies that hinder the well-being of their patients. The intensity in engagement can range from providing data on the impact of policies on health to policy leaders, to collaborating with community-organizations to enact policy changes. All engagement should center on health equity.

### BPS+R Model

**Figure 1:**
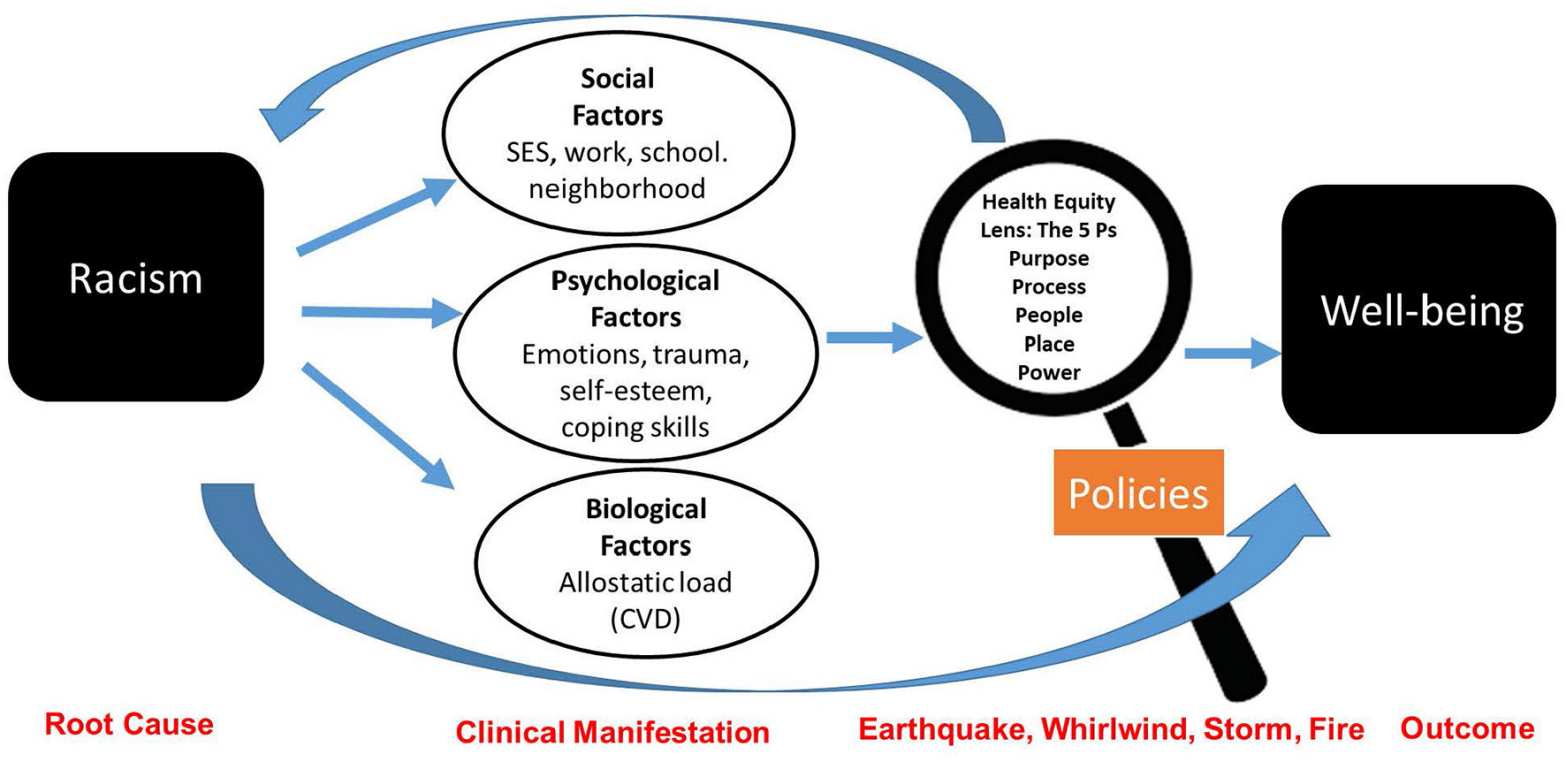
Biopsychosocial model: addressing racism through a health-equity lens.

We propose the BPS+R model that incorporates the 5Ps Health Equity and Empowerment Lens (26). The 5Ps (purpose, people, place, process and power) can be used for both institutional and civic engagement outside the clinical encounter. The 5 Ps are a set of guiding principles and reflective questions to evaluate whether policies have a positive impact on well-being and achieve health equity Figure 1. For example, racialized segregation created by federal housing policies has had a lasting impact not only on where a person lives, but also their access to employment and exposure to health hazards (e.g., pollution) (28). These downstream inequities manifest in a person's biological, psychological and social health. Therefore, use of the BPS model alone will be ineffective in treating the root cause of poor health, racism. Treatment can be achieved through changes in policies and interpersonal work. These principles have been endorsed as a training tool by several county health departments to help develop more racially equitable policies and programs (29–32).

The 5Ps can be used to assess whether any proposed changes in policy further exacerbate racism or work toward health equity. Consider Mr. Thomas, a (fictional) 55 yr. old man with uncontrolled hypertension, who lives alone in his childhood home. The home is paid for, but he has to work two jobs to keep up with the property taxes and amenities. The neighborhood has changed since he was a child. Gentrification and poverty have changed the social and physical composition of the neighborhood. He cannot afford, nor does he want to leave the home his parents struggled to pay for in the 1970s. Table 1 outlines a composite example of how trainees can treat the root cause of Mr. Thomas' uncontrolled blood pressure. While it may appear that treating his uncontrolled blood pressure with medication will solve the issue, a deeper reflection reveals the contrary. A lack of institutional resources and racialized gentrification are what need to be treated.

**Table 1:** Example of how trainees can treat the root cause of uncontrolled blood pressure.

	**Issue**	**Clinical Action:** **BPS Model Tx only**	**Institutional Action:** **BPS Model Tx +QI HE**	**Civic Action:** **BPS Model Tx + 5Ps HE lens**
Biological	Mr. Thomas' blood pressure has been uncontrolled for over a year.	Rx: HTN meds and recommend that he monitor his blood pressure at home.	Ask health care leadership to purchase a set of digital home blood pressure monitors (HBPM) that patients like Mr. Thomas can borrow for free as they work to bring their BP in control. **P**urpose: To eliminate the impact of racism on blood pressure control. **P**lace: How are blood pressure control tools distributed amongst patients within your practice? **P**rocesses: What institutional policies contribute to Mr. Thomas' inequities? Is there support for the patient without broadband access that the patient would like to participate in for HBPM? **P**ower: How have you helped to shift power dynamics to better integrate voices and priorities of Mr. Thomas?	Donate time and or financial resources to local a community-based organization that is working on the issue of walkable sidewalks and ask your colleagues to do the same. **P**urpose: To eliminate the impact of racism on built neighborhood. **P**eople: Ensure the policy action will positively affect your patients. **P**lace: To make certain public resources are being equitably distributed geographically. **P**rocesses: Check and re-check whether the policy will inadvertently contribute to Mr. Thomas' inequities. **P**ower: Confirm you have helped shift the power dynamics to better integrate voices and priorities of Mr. Thomas.

Mr. Thomas is a composite of patients we worked with; real-world Mr. Thomas' are not difficult to find. Therefore, the BPS+R model is critical to reducing health disparities. Without it, trainees lack the sociocultural context that explains the circumstances that have led to the health outcomes and health behaviors of many BIPOC patients.

### Comparison to Other Anti-racism Training Models

The BPS-R is unique in several respects. First, it is grounded in the influential BPS model (33), while adding the critical lens of race. Second, the model integrates existing approaches to antiracism training. A realist review of anti-racist pedagogy in health professional education distinguished four pedagogical approaches: dialogue across social groups, deconstructing power and privilege, trainee transformation and application to practice (34). Our model integrates these four approaches. It adds the race lens to promote dialogue across race. It deconstructs power and privilege using the 5 P's. It promotes trainee transformation through the mini-ethnography of the BPS combined with discussion and reflection.

## Limitations

There are a few limitations to our proposed model we would like to note. First, we recognize that broader structural changes and training will be required to enhance the utility of the BPS-R model. Anti-racism training is new to medical education. Similar to other existing models, there is a need for research to assess the impact of BPS-R training on trainees, patients, and communities (35). Future research will need to delineate the specific strengths and weaknesses of the BPS-R and other antiracism training models. Second, the 5Ps is a pragmatic, reflective model that trainees can use as a lens to engage in civic action and it is not a panacea for eliminating the impact of racism on patients. Nonetheless, there is precedent in our community for health professionals successfully engaging in civic action to drive health policy change (36–39). The *Rochester Lead Law* (38, 40–42) is an example of a public policy that was led by members of the community (including health care professionals), which has helped to reduce lead poisonings and focus resources to our most economically challenged neighborhoods. This level of civic engagement is not trivial and requires personal will, courage, and collective power (43). But, as Hardeman et al. note, clinicians and researchers wield power, privilege, and responsibility for dismantling structural racism (44).

### Lessons

Our department has incorporated the role of racism into the curriculum in various ways over several decades, often through community medicine and through the biopsychosocial curriculum. Our experiences suggest that incorporating anti-racism into the BPS model takes time. Residents vary in their willingness to acknowledge their own implicit biases and White privilege. Ensuring consistency in racial diversity among trainees has been a challenge. Peers are an important source of learning for residents (45), and lack of diversity fosters color blindness.

Similarly, leadership matters. Both our medical school and departmental leaders have made public commitments to advancing racial equity and inclusion and implementing anti-racism efforts. Leaders can help keep anti-racism in the spotlight and foster a culture of learning and reflecting. Applying an antiracism lens to the BPS model is a reasonable place to start.

## Data Availability Statement

The original contributions presented in the study are included in the article/supplementary material, further inquiries can be directed to the corresponding author.

## Author Contributions

All authors listed have made a substantial, direct and intellectual contribution to the work, and approved it for publication.

## Conflict of Interest

The authors declare that the research was conducted in the absence of any commercial or financial relationships that could be construed as a potential conflict of interest.

## Publisher's Note

All claims expressed in this article are solely those of the authors and do not necessarily represent those of their affiliated organizations, or those of the publisher, the editors and the reviewers. Any product that may be evaluated in this article, or claim that may be made by its manufacturer, is not guaranteed or endorsed by the publisher.
